# Hog1-mediated stress tolerance in the pathogenic fungus *Trichosporon asahii*

**DOI:** 10.1038/s41598-023-40825-y

**Published:** 2023-08-19

**Authors:** Yasuhiko Matsumoto, Yu Sugiyama, Tae Nagamachi, Asami Yoshikawa, Takashi Sugita

**Affiliations:** Department of Microbiology, Meiji Pharmaceutical University, 2-522-1, Noshio, Kiyose, Tokyo 204-8588 Japan

**Keywords:** Cell biology, Genetics, Microbiology, Pathogenesis

## Abstract

*Trichosporon asahii* is an opportunistic pathogenic fungus that causes severe and sometimes fatal infections in immunocompromised patients*.* Hog1, a mitogen-activated protein kinase, regulates the stress resistance of some pathogenic fungi, however its role in *T. asahii* has not been investigated. Here, we demonstrated that the *hog1* gene-deficient *T. asahii* mutant is sensitive to high temperature, cell membrane stress, oxidative stress, and antifungal drugs. Growth of the *hog1* gene-deficient *T. asahii* mutant was delayed at 40 °C. The *hog1* gene-deficient *T. asahii* mutant also exhibited sensitivity to sodium dodecyl sulfate, hydrogen peroxide, menadione, methyl methanesulfonate, UV exposure, and antifungal drugs such as amphotericin B under a glucose-rich condition. Under a glucose-restricted condition, the *hog1* gene-deficient mutant exhibited sensitivity to NaCl and KCl. The virulence of the *hog1* gene-deficient mutant against silkworms was attenuated. Moreover, the viability of the *hog1* gene-deficient mutant decreased in the silkworm hemolymph. These phenotypes were restored by re-introducing the *hog1* gene into the gene-deficient mutant. Our findings suggest that Hog1 plays a critical role in regulating cellular stress responses in *T. asahii*.

## Introduction

The pathogenic fungus *Trichosporon asahii* is a basidiomycete yeast often isolated from soil, human blood, sputum, skin, feces, and urine^1–6^. *T. asahii* causes severe fungal infections in immunocompromised patients^7,8^, and the mortality rate of deep mycoses caused by *T. asahii* is higher than that of infections caused by *Candida albicans*, another pathogenic fungus (80% vs. 40%)^9^. Micafungin, an echinocandin antifungal drug, is used to treat for patients with suspected fungal infections. If infections are caused by *T. asahii*, severe infections develop because *T. asahii* is tolerant to micafungin^10,11^. Amphotericin B- and azole-resistant *T. asahii* strains have also been isolated from patients^12,13^. Therefore, infections caused by *T. asahii* are problematic in clinical settings^8^.

Hog1-mediated signaling pathway is involved in fungal resistance to several types of stressors^14–16^. The Hog1 protein, a mitogen-activated protein kinase (MAPK), translocates from the cytoplasm to the nucleus in response to environmental stress and acts in conjunction with multiple transcription factors to regulate the expression of genes related to stress resistance^15,16^. Hog1-mediated stress resistance and gene regulation also affect the virulence of the pathogenic fungus *Cryptococcus neoformans*^17–19^. The Hog1 protein is also involved in the pathogenicity of *C. albicans* to the host by increasing its resistance to various stressors and controlling the morphological change from yeast to mycelium^20–24^. Moreover, stress response systems are related to morphological changes in *C. albicans*^25–28^. However, the role of Hog1 in stress tolerance and virulence of *T. asahii* remains unknown.

To elucidate the molecular mechanisms underlying *T. asahii* virulence, a silkworm infection model and a method for constructing a gene-deficient *T. asahii* mutant were established^29,30^. The use of an invertebrate silkworm model for infection experiments is highly advantageous because silkworms are less costly to rear in large numbers, and fewer ethical problems are associated with their use^31,32^. The virulence of *T. asahii* clinical isolates can be evaluated quantitatively by calculating the half-maximal lethal dose (LD_50_), which is the dose of a pathogen required to kill half of the animals in a group^29^. An efficient gene-targeting system was developed to generate gene-deficient mutants of *T. asahii*^30,33^. Gene-deficient *T. asahii* mutants of calcineurin, a calcium-calmodulin-activated phosphatase, exhibit decreased virulence against silkworms^34^. These findings demonstrate that an evaluation system comprising a silkworm infection model and a gene manipulation system is useful for elucidating the molecular mechanisms of *T. asahii* infection.

In the present study, we generated a *hog1* gene-deficient *T. asahii* mutant and characterized the phenotypes related to stress resistance and virulence in a silkworm infection model. The *hog1* gene-deficient mutant exhibited sensitivity to compounds known to cause membrane damage and to antifungal drugs. Furthermore, the virulence of the *hog1* gene-deficient *T. asahii* mutant against silkworms decreased. Our findings suggest that the virulence and cellular stress responses of *T. asahii* are controlled by the Hog1 mediated-signaling pathway.

## Results

### Conservation of the amino acid sequence of *T. asahii* Hog1 protein among representative fungi

**Figure 1:**
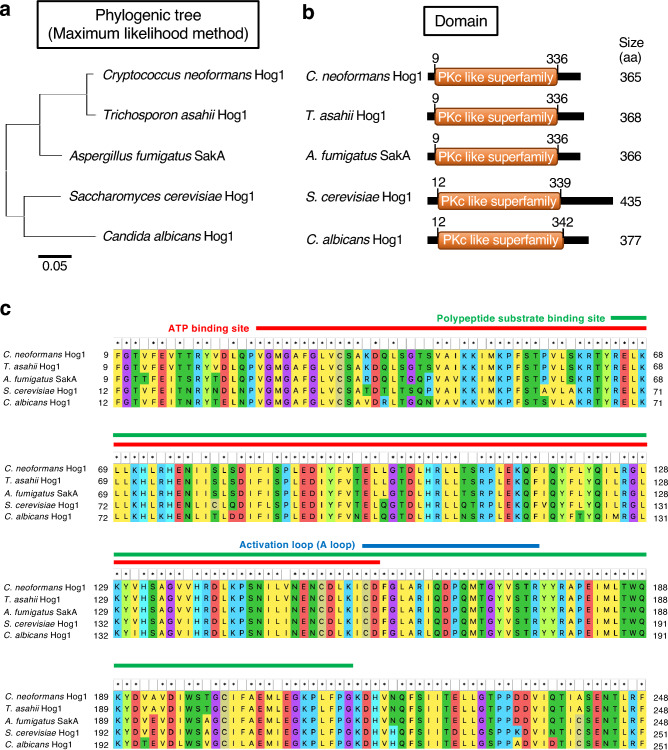
Estimation of Hog1 protein in *T. asahii* and conservation of the amino acids among typical fungi. (**a**) Phylogenetic tree of *T. asahii*, *C. neoformans*, *S. cerevisiae*, *C. albicans* Hog1, and *A. fumigatus* SakA was generated according to the maximum likelihood method. (**b**) Estimation of the functional domain of *T. asahii* Hog1 and homologs. (**c**) Conservation of the amino acids of protein kinase c-like superfamily domain in *T. asahii* Hog1. ATP binding site, polypeptide substrate binding site, and activation loop (A loop) are indicated red, green, and blue lines, respectively.

We performed an amino acid sequence homology analysis with the Hog1 protein of *T. asahii* based on genomic information from a representative model fungus, *Saccharomyces cerevisiae*, and representative pathogenic fungi, *Aspergillus fumigatus*, *C. neoformans*, and *C. albicans*. The amino acid sequence of the Hog1 protein of *T. asahii* was more than 70% identical to those of these fungi (Fig. 1a and Supplementary Table S1). The Hog1 protein of *T. asahii* contains a protein kinase c-like superfamily domain, which includes an ATP-binding site, polypeptide substrate-binding site, and activation loop (Fig. 1b,c, and Supplementary Table S2).

### Generation of the *hog1* gene-deficient mutant and the gene-reintroduced strain

**Figure 2:**
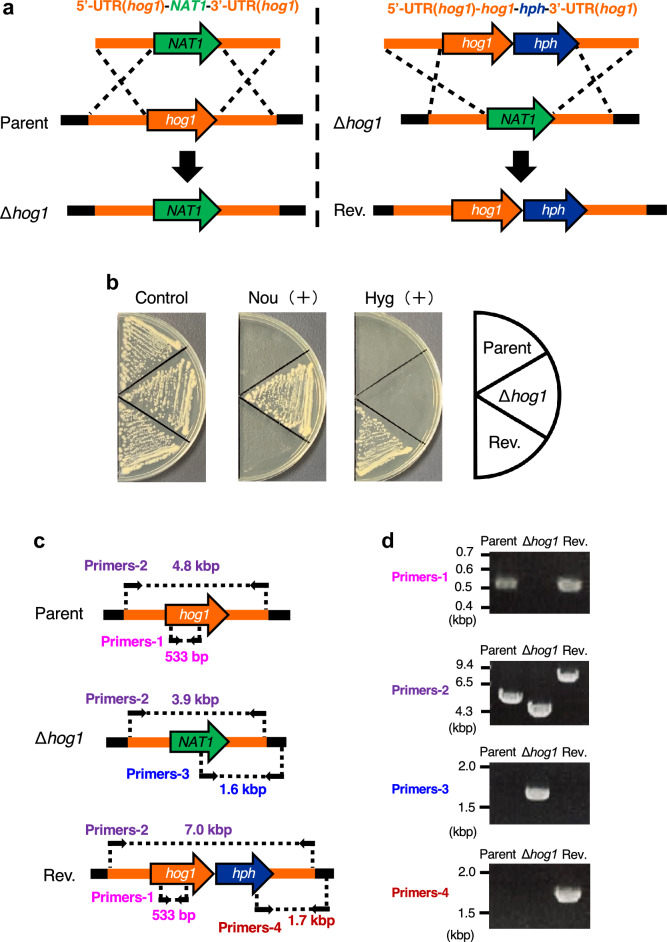
Generation of the *hog1* gene-deficient *T. asahii* mutant and its revertant. (**a**–**d**) Generation of the *hog1* gene-deficient *T. asahii* mutant and its revertant. (**a**) Strategy for generating the *hog1* gene-deficient mutant (∆*hog1*) and its revertant (Rev.). Predicted genomes of the *hog1* gene-deficient mutant and its revertant are shown. (**b**) The parent strain (Parent), *hog1* gene-deficient mutant (∆*hog1*), and its revertant (Rev.) were spread on SDA with nourseothricin (Nou) (100 µg/mL) or hygromycin B (Hyg) (100 µg/mL) and incubated at 27 °C for 2 days. (**c**) Location of the primers for confirming the genome structure of the *hog1* gene-deficient candidate by PCR using extracted genome DNA. (**d**) Confirmation of the genotypes of the *hog1* gene-deficient mutant (∆*hog1*) and its revertant (Rev.) by PCR using extracted genome DNA. Cropped blots were used. Full-length blots are presented in Supplementary Fig. S1.

The *ku70* gene-deficient strain of *T. asahii* MPU129 has a high homologous recombination efficiency, making it useful as a parent strain^30^. Using this strain, we generated the *hog1* gene-deficient *T. asahii* mutant. The targeting DNA fragment used to generate the *hog1* gene-deficient mutant contains the *NAT1* gene, which leads to nourseothricin resistance (Fig. 2a). Transformants with nourseothricin resistance were obtained by introducing the DNA fragment through electroporation (Fig. 2b). Genomic DNA was extracted from the transformants and replacement of the *hog1* gene was confirmed by polymerase chain reaction (PCR) (Fig. 2c,d). Secondary genetic mutations such as point mutations may occur during the generation of gene-deficient mutants. Therefore, we also generated a revertant strain of the *hog1* gene-deficient mutant to confirm that the phenotype of the gene-deficient strain is due to a deficiency of the targeted gene. The targeting DNA fragment used to generate the revertant strain of the *hog1* gene-deficient mutant contains the *hph* gene, which leads to hygromycin B resistance (Fig. 2a). Transformants with hygromycin B resistance were obtained by introducing the DNA fragment through electroporation (Fig. 2b). Reintroduction of the *hog1* gene was verified by PCR using DNA extracted from the transformants (Fig. 2c,d). These results confirmed the generation of the *hog1* gene-deficient *T. asahii* mutant and the *hog1* gene-reintroduced revertant.

### Role of the *hog1* gene in the growth of *T. asahii* under high temperature

**Figure 3:**
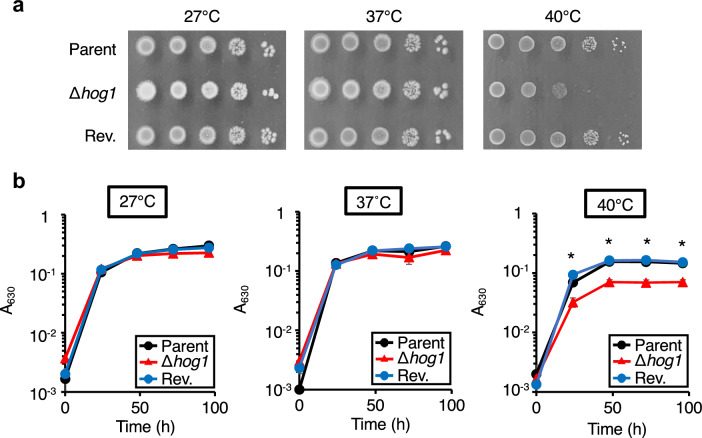
Temperature sensitivity of the *hog1* gene-deficient *T. asahii* mutant. (**a**) The *T. asahii* parent strain (Parent), the *hog1* gene-deficient mutant (∆*hog1*), and the revertant of ∆*hog1* (Rev.) were grown on SDA and incubated at 27 °C for 1 day. *T. asahii* cells were suspended in physiologic saline solution and filtered through a 40-μm cell strainer. Series of tenfold dilution of the fungal suspension were prepared using saline. Five microliters of each cell suspension was spotted on the SDA. Agar plates were incubated at 27 °C, 37 °C, or 40 °C for 24 h. (**b**) The *T. asahii* parent strain (Parent), the *hog1* gene-deficient mutant (∆*hog1*), and the revertant of ∆*hog1* (Rev.) were inoculated on Sabouraud medium and incubated at 27 °C, 37 °C, or 40 °C. Absorbance of the culture at 630 nm was monitored. Data are shown as means ± standard error of the mean (SEM). Statistically significant differences between groups were evaluated using Tukey’s test. *P < 0.05.

In *C. neoformans* and *S. cerevisiae*, the growth of *hog1* gene-deficient strains is delayed at 39–40 °C compared to their respective parents^14,17,35^. We examined whether Hog1 was involved in the high-temperature resistance of *T. asahii*. Growth of the *hog1* gene-deficient *T. asahii* mutant was delayed at 40 °C (Fig. 3). On the other hand, there was no delay in the growth of the *hog1* gene-deficient mutant at 27 °C or 37 °C (Fig. 3). The high-temperature-sensitive phenotype of the *hog1* gene-deficient mutants was suppressed in the revertant strain (Fig. 3). These results suggest that *hog1* gene-deficient *T. asahii* mutant is sensitive to high-temperature stress.

### Functions of the *hog1* gene in the stress resistance of *T. asahii*

**Figure 4:**
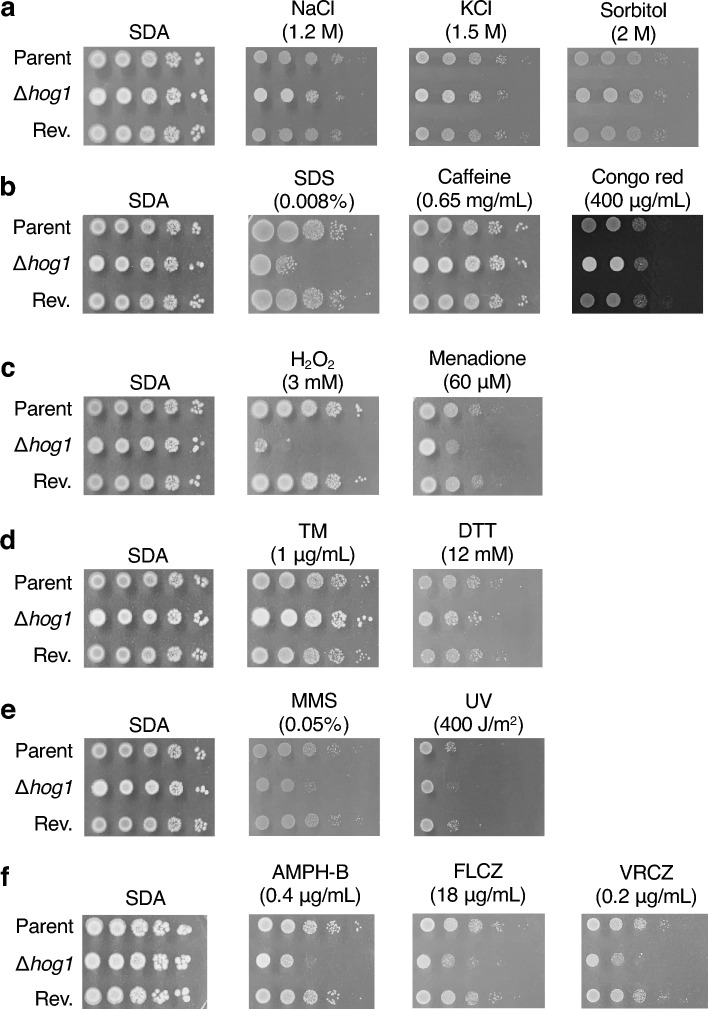
Sensitivity of the *hog1* gene-deficient *T. asahii* mutant against stressors. (**a**–**f**) The *T. asahii* parent strain (Parent), the *hog1* gene-deficient mutant (∆*hog1*), and the revertant of ∆*hog1* (Rev.) were grown on SDA and incubated at 27 °C for 1 day. *T. asahii* cells were suspended in physiologic saline solution. Series of tenfold dilution of the fungal suspension were prepared using saline. Five microliters of each cell suspension was spotted on the SDA containing NaCl (1.2 M), KCl (1.5 M), sorbitol (2 M), SDS (0.008%), caffeine (0.65 mg/mL), Congo red (400 µg/mL), H_2_O_2_ (3 mM), menadione (60 µM), tunicamycin (TM, 1 µg/mL), dithiothreitol (DTT, 12 mM), methyl methanesulfonate (MMS, 0.05%), amphotericin-B (AMPH-B, 0.4 µg/mL), fluconazole (18 µg/mL), and voriconazole (VRCZ, 0.2 µg/mL). Cells on SDA were exposed to UV (400 J/m^2^). Each agar plate of (**a**–**d**) or (**f**) was incubated at 27 °C for 24 h or 48 h, respectively.

Next, we examined whether Hog1 contributes to the tolerance to several stresses in *T. asahii*. NaCl, KCl and sorbitol causes osmotic stress^20–24,36,37^. Sodium dodecyl sulfate (SDS) damages the cell membrane^35,38–40^. Congo red and caffeine cause cell wall damage^38,40^. Hydrogen peroxide (H_2_O_2_) induces oxidative stress^17,19,20,23,41,42^. Tunicamycin (TM) and dithiothreitol (DTT) induce endoplasmic reticulum (ER) stress^24,35,43^. UV exposure and methyl methanesulfonate (MMS) cause DNA damage^17,35,44^. The number of tolerant cells in the *hog1* gene-deficient *T. asahii* mutant was decreased on Sabouraud dextrose agar (SDA) containing SDS, H_2_O_2_, menadione, MMS, or upon UV exposure, but not on SDA containing NaCl, KCl, sorbitol, caffeine, Congo red, TM, or DTT (Fig. 4a–e).

Amphotericin B and fluconazole are used to treat *T. asahii* infections^45,46^. The *hog1* gene-deficient mutants of *C. neoformans* and *C. albicans* are sensitive to an antifungal agent the amphotericin B (AMPH-B)^19,23,38,42^. The *hog1* gene-deficient mutant of *C. albicans* is susceptible to azole antifungals, whereas the *hog1* gene-deficient mutant of *C. neoformans* is resistant^19,23,38,42^. The number of tolerant cells in the *hog1* gene-deficient *T. asahii* mutant was decreased on SDA containing AMPH-B, fluconazole (FLCZ), or voriconazole (VRCZ) (Fig. 4f). Moreover, we determined the MIC values of antifungal drugs for inhibiting the growth of the parent strain or *hog1* gene-deficient *T. asahii* mutant. Compared to the parental strain, the MIC values of AMPH-B, FLCZ, ITCZ, and VRCZ were lower in the *hog1* gene-deficient *T. asahii* mutant (Table 1). The phenotypes of the *hog1* gene-deficient mutant were suppressed in the revertant. These findings suggest that the *hog1* gene in *T. asahii* is involved in resistance to cell membrane damage, oxidative stress, DNA damage, and antifungal drugs, such as AMPH-B and azole antifungals.

**Table 1:** MIC values of antifungal drugs against *T. asahii* strains.

MIC (µg/mL)	Parent	∆*hog1*	Hog1 (revertant)
MCFG	> 16	> 16	> 16
CPFG	16–> 16	16	16
AMPH-B	2	0.25–0.5	2
5-FC	> 64	> 64	> 64
FLCZ	8	1–2	8–16
ITCZ	0.5–1	0.12–0.25	0.5
VRCZ	0.12–0.25	0.03–0.06	0.12
MCZ	0.5–1	0.25–0.5	1

### Involvement of the *hog1* gene in the stress resistance of *T. asahii* under a glucose-restricted condition

**Figure 5:**
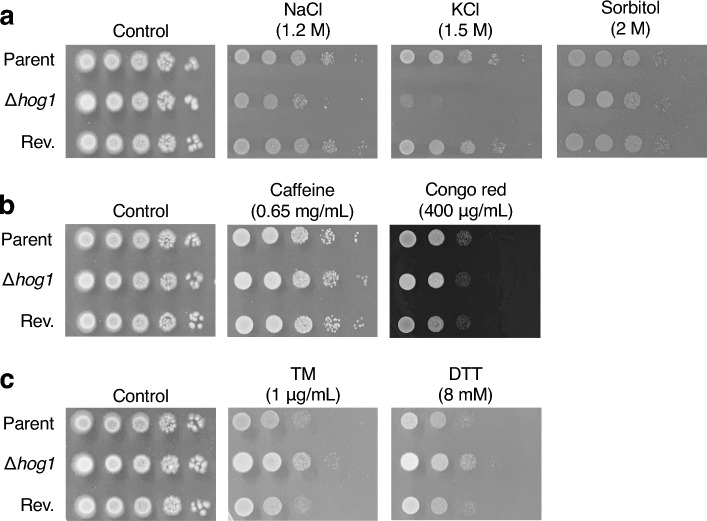
Effect of glucose on sensitivity of the *hog1* gene-deficient mutants against stressors. The *T. asahii* parent strain (Parent), the *hog1* gene-deficient mutant (∆*hog1*), and the revertant of ∆*hog1* (Rev.) were grown on glucose-limited peptone agar and incubated at 27 °C for 1 day. *T. asahii* cells were suspended in physiologic saline solution. Series of tenfold dilution of the fungal suspension were prepared using saline. Five microliters of each cell suspension was spotted on the glucose-restricted peptone agar containing NaCl (1.2 M), KCl (1.5 M), sorbitol (2 M), caffeine (0.65 mg/mL), Congo red (400 µg/mL), tunicamycin (TM, 1 µg/mL), or dithiothreitol (DTT, 8 mM). Each agar plate was incubated at 27 °C for 48 h.

The *hog1* gene-deficient mutant of *T. asahii* did not exhibit clear sensitivity to KCl, NaCl, sorbitol, caffeine, Congo red, TM, or DTT under a glucose-rich condition. Hog1 in *C. neoformans* plays a pivotal role in the regulation of stress responses under a glucose-restricted condition^47^. Therefore, we investigated whether *hog1* gene deficiency in *T. asahii* causes sensitivity to these compounds under a glucose-restricted condition. Under a glucose-restricted condition, the growth of the *hog1* gene-deficient *T. asahii* mutant was delayed by NaCl and KCl, but not by sorbitol, caffeine, Congo red, TM, or DTT (Fig. 5). The phenotypes of NaCl and KCl sensitivity of the *hog1* gene-deficient mutant were suppressed in the revertant (Fig. 5). These findings suggest that the *hog1* gene in *T. asahii* contributes to the stress caused by NaCl and KCl under a glucose-restricted condition.

### Role of the *hog1* gene in hyphal formation by *T. asahii*

**Figure 6:**
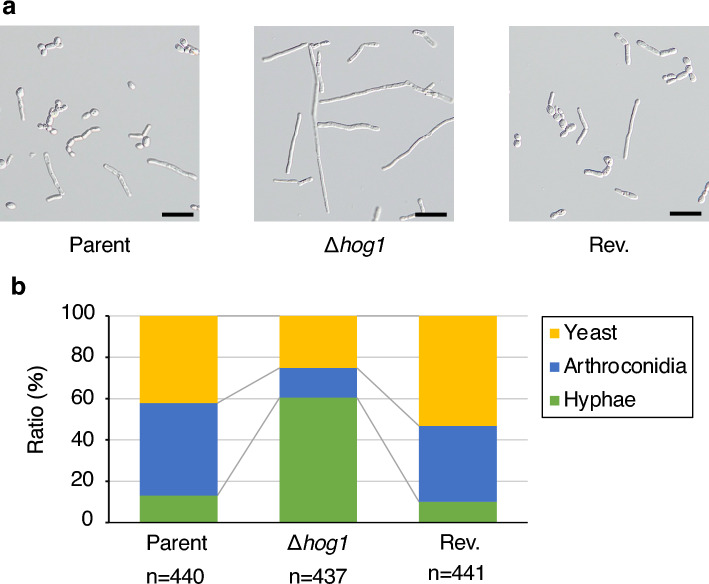
Effect of *hog1* gene-deficiency on *T. asahii* morphology in Sabouraud dextrose medium. The *T. asahii* parent strain (Parent), the *hog1* gene-deficient mutant (∆*hog1*), and the revertant of ∆*hog1* (Rev.) were grown on SDA and incubated at 27 °C for 2 days. *T. asahii* cells were suspended in physiologic saline solution and filtered through a 40-μm cell strainer. Absorbance at 630 nm of the *T. asahii* cell suspension was adjusted to 1. The cell suspension (10 µL) was added to Sabouraud dextrose medium (100 µL). The solutions were incubated at 27 °C for 48 h. The incubated solution was placed on glass slides and covered by glass coverslips. (**a**) Samples were examined with bright light under a microscope. Scale bar, 20 µm. (**b**) The pictures were randomly obtained. The numbers of 3 cell types: yeast, arthroconidia, and hyphae, were counted.

*T. asahii* has several morphological forms, including yeast, hyphae (filament form), and arthroconidia (chains of cells and asexual spores)^4^. In *C. albicans*, Hog1 is a key factor that regulates hyphal formation^20,21,23,25–28,48^. We examined whether *hog1* gene deficiency affects hyphal formation in *T. asahii*. In Sabouraud dextrose medium, the hyphae ratio was higher in the *hog1* gene-deficient mutant than in the parental strain (Fig. 6). On the other hand, the yeast and arthroconidia ratio were lower in the *hog1* gene-deficient mutant than in the parental strain (Fig. 6). The hyphae per yeast ratios in the parental strain and the *hog1* gene-deficient mutant were 0.3 and 2.3, respectively. The phenotype of higher hyphal-forming activity in the *hog1* gene-deficient mutant was suppressed in the revertant (Fig. 6). These observations suggest that *hog1* gene regulates hyphal formation by *T. asahii*.

### Hog1-dependency of *T. asahii* virulence against silkworms

**Figure 7:**
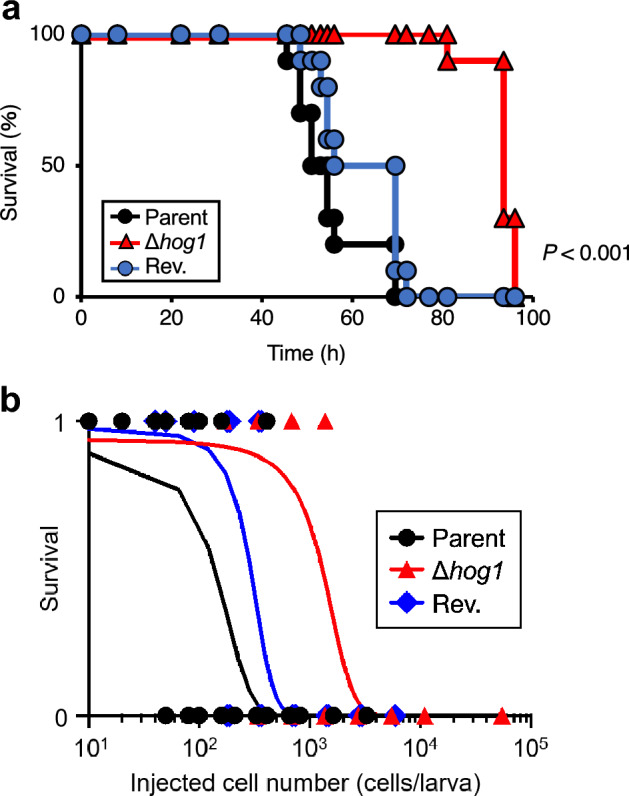
Attenuated virulence of the *hog1* gene-deficient *T. asahii* mutant against silkworms. (**a**) The *T. asahii* parent strain (Parent; 1.3 × 10^3^ cells/larva), the *hog1* gene-deficient mutant (∆*hog1*; 1.6 × 10^3^ cells/larva), and the revertant from ∆*hog1* (Rev.; 1.1 × 10^3^ cells/larva) were injected into the silkworm hemolymph and the silkworms were incubated at 37 °C. Silkworm survival was monitored for 96 h. The significance of differences between the parent strain group and the *hog1* gene-deficient mutant group was calculated by the log-rank test based on the curves by the Kaplan–Meier method. *P* < 0.05 was considered significant. n = 10/group. (**b**) Number of surviving silkworms at 37 °C was determined at 72 h after administration of the fungal cells (1 × 10^2^ to 5.5 × 10^4^ cells/larva) into the silkworm hemolymph. Surviving and dead silkworms are indicated as 1 and 0, respectively. n = 4/group. Curves were drawn from combined data of 2 independent experiments by a simple logistic regression model. The result of the half-maximal lethal time (LT_50_), which is the time required to kill half of the animals in a group, were shown in the Supplementary Fig. S2.

We examined whether a deficiency of the *hog1* gene reduced the virulence of *T. asahii* against silkworms. The survival time of silkworms injected with the *hog1* gene-deficient mutant was longer than that of the parental strain (Fig. 7a). The LD_50_ value of the *hog1* gene-deficient mutant was tenfold higher than that of the parent strain (Fig. 7b, and Table 2). This phenotype was suppressed in the revertant (Fig. 7 and Table 2). These findings suggest that the *hog1* gene is involved in the virulence of *T. asahii* against silkworms.

**Table 2:** LD_50_ values of *T. asahii* strains.

*T. asahii* strains	LD_50_ (cells/larva)
Parent strain	140
∆*hog1*	1300
Hog1 (Revertant)	300

### Decreased viability of the *hog1* gene-deficient *T. asahii* mutant in silkworm hemolymph

**Figure 8:**
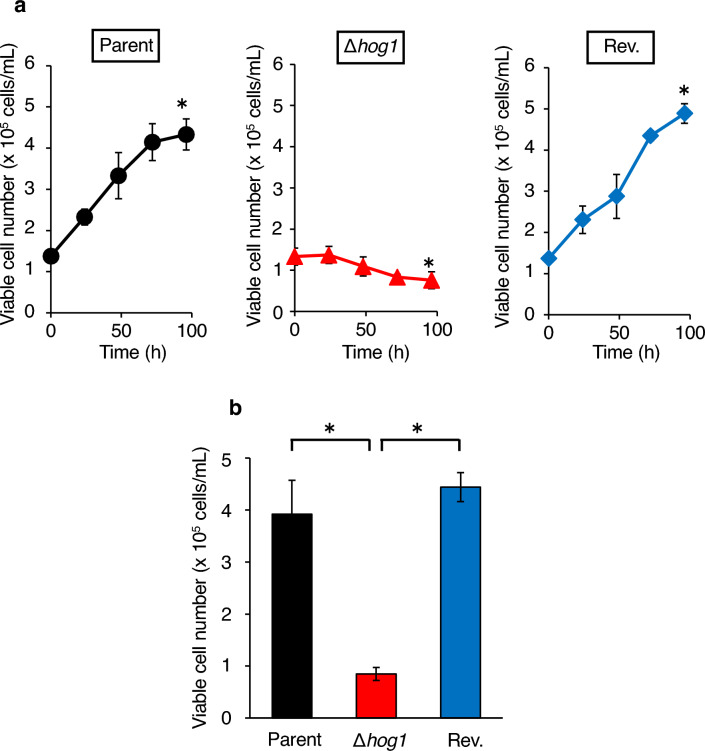
Decrease in number of viable cells of the *hog1* gene-deficient *T. asahii* mutant in harvested silkworm hemolymph. The *T. asahii* parent strain (Parent), the *hog1* gene-deficient mutant (∆*hog1*), and the revertant of ∆*hog1* (Rev.) were grown on SDA and incubated at 27 °C for 1 day. *T. asahii* cells were suspended in physiologic saline solution and filtered through a 40-μm cell strainer. Absorbance at 630 nm of the *T. asahii* cell suspension was adjusted to 1–1.5. The cell suspension (10 µL) was added to harvested silkworm hemolymph (90 µL) and the solutions were incubated at 37 °C for 4 days. The *T. asahii* viable cells were determined by the CFU method. (**a**) Time course experiment. n = 3/group. Statistically significant differences between 0 and 4 days were evaluated using Student’s *t*-test. *P < 0.05. (**b**) *T. asahii* viable cells were determined at 4 days after inoculation. Statistically significant differences between groups were evaluated using Tukey’s test. *P < 0.05.

**Figure 9:**
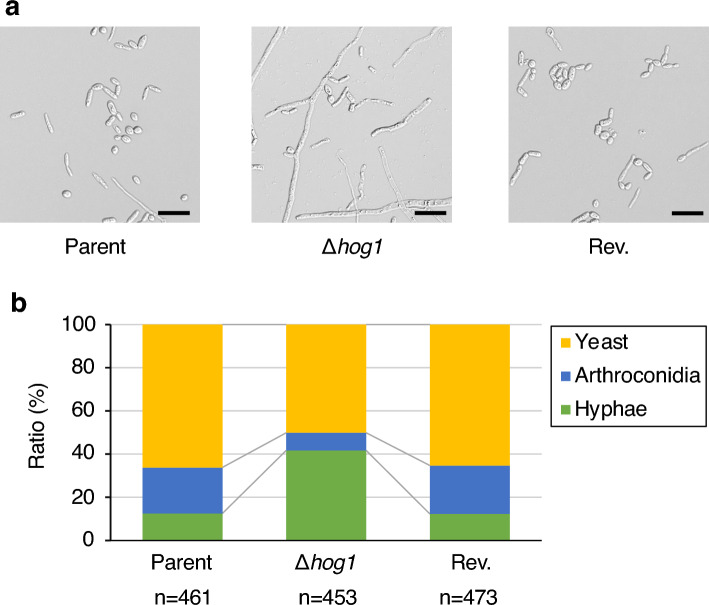
Role of *hog1* gene on *T. asahii* morphological change in silkworm hemolymph. The *T. asahii* parent strain (Parent), the *hog1* gene-deficient mutant (∆*hog1*), and the revertant of ∆*hog1* (Rev.) were grown on SDA and incubated at 27 °C for 2 days. *T. asahii* cells were suspended in physiologic saline solution and filtered through a 40-μm cell strainer. Absorbance at 630 nm of the *T. asahii* cell suspension was adjusted to 1. The cell suspension (10 µL) was added to hemolymph (100 µL). The solutions were incubated at 27 °C for 48 h. The incubated solution was placed on glass slides and covered by glass coverslips. (**a**) Samples were examined with bright light under a microscope. Scale bar, 20 µm. (**b**) The pictures were randomly obtained. The numbers of 3 cell types: yeast, arthroconidia, and hyphae, were counted.

We examined the effect of *hog1* gene deficiency on *T. asahii* cell viability in harvested silkworm hemolymph in vitro. In the parent strain, the number of viable cells in the silkworm hemolymph increased after incubation (Fig. 8a). On the other hand, the viable cell number of the *hog1* gene-deficient mutant decreased in the silkworm hemolymph after 96 h of incubation (Fig. 8a). The viable cell number of the *hog1* gene-deficient mutant in the silkworm hemolymph after 96 h of incubation was lower than that of the parental strain (Fig. 8b). In the silkworm hemolymph, the hyphae ratio was higher in the *hog1* gene-deficient mutant than in the parental strain (Fig. 9). The phenotypes of higher hyphal-forming activity in the *hog1* gene-deficient mutant were suppressed in the revertant (Figs. 8 and 9). This observation suggests that the *hog1* gene is involved in the morphological changes of *T. asahii* in the silkworm hemolymph and tolerance against the factors in silkworm hemolymph.

## Discussion

In the present study, we generated a *hog1* gene-deficient *T. asahii* mutant to examine its role of the *hog1* gene on stress tolerance and virulence. The *hog1* gene in *T. asahii* plays a vital role in stress tolerance to high-temperature, oxidative agents, antifungal drugs, and factors in the silkworm hemolymph. Our findings suggest that the stress tolerance of *T. asahii* is regulated by the Hog1-mediated signaling pathway and that the stress tolerance of *T. asahii* might contribute to its virulence.

Hog1 is a MAPK that is widely conserved in fungi^16,49^. In fungi such as *S. cerevisiae* and *C. neoformans*, the Hog1-mediated signaling pathway is involved in resistance to various stressors^15,16^. A gene encoding a protein with more than 70% homology to the amino acid sequence of Hog1 in *S. cerevisiae* and *C. neoformans* was identified in the genome of *T. asahii*. Furthermore, Hog1 protein in *T. asahii* contains a phosphorylation domain. Therefore, we assumed that Hog1 is also involved in the kinase cascade related to the stress responses in *T. asahii*.

The *hog1* gene is required for the normal growth of *T. asahii* under a high-temperature condition (40 °C). Severe growth inhibition of the *hog1* gene-deficient *T. asahii* mutant was observed at 40 °C compared with that at 27 °C and 37 °C. Therefore, the high-temperature stress response in *T. asahii* is mediated by Hog1. In *C. neoformans* and *S. cerevisiae*, the Hog1-mediated signaling pathway is required for growth at 40 °C^14,17,35^. *S. cerevisiae* Hog1 activates by high temperature and is required for immediate recovery from high temperature conditions^14^. Moreover, high temperature stress simultaneously activates Hog1 and the cell wall integrity pathway in *S. cerevisiae*^50^. In *Candida albicans*, Hog1 regulates the cell wall integrity pathway^51^. Deletion of the *CEL1* gene, which encodes a lytic polysaccharide monooxygenase involved in cell wall integrity in *Cryptococcus neoformans*, causes susceptibility to high temperature stress^52^. Therefore, we assumed that Hog1 in *T. asahii* may be activated by high temperature stress and regulates the cell wall integrity pathway for high temperature stress tolerance.

Hog1 is required for several different types of stress responses in *T. asahii*. Hog1 in *S. cerevisiae* and *C. albicans*, and SakA in *A. fumigatus* play important roles in osmotic stress responses^20–24,36,37^. In *C. neoformans*, Hog1 is required for the resistance to stress caused by NaCl and KCl under a glucose-restricted condition^19,47^. In *T. asahii*, Hog1 is not involved in osmotic stress responses under a glucose-rich condition. On the other hand, Hog1 in *T. asahii* is related to resistance to NaCl and KCl under a glucose-restricted condition. The phenotypes of *T. asahii* are similar to those of *C. neoformans*. Upstream of the Hog1 MAPK signaling pathway in *S. cerevisiae* are two branching cascades consisting of Sho1 or Sln1, which phosphorylate Pbs2 MAPK kinase kinase^14,15^. The gene encoding the Sln1 homolog of *S. cerevisiae* is not present in the *C. neoformans* genome, however, and histidine kinase Tco2, instead of Sln1, acts as an osmotic sensor^15,19,53^. The histidine kinase in *T. asahii* has 46% amino acid sequence homology with *C. neoformans* Tco2. Moreover, no protein in *T. asahii* has at least 30% amino acid sequence homology with the osmotic sensor Sho1 in *S. cerevisiae*. Therefore, *T. asahii* may have a Tco2-mediated response mechanism to osmotic stress similar to that of *C. neoformans*. In *C. neoformans*, Ena1, a Na+/ATPase regulated by Hog1, exports cations such as sodium and potassium ions^47,54^. The *ena1* gene-deficient *C. neoformans* mutant exhibits similar growth as the wild-type strain under stress induced by NaCl and KCl in glucose-rich conditions, but shows susceptibility under low-glucose conditions^47,54^. In *T. asahii*, Hog1 may regulate the stress tolerance mechanism for sodium and potassium ions mediated by Ena1 under low-glucose conditions. Further studies are needed to clarify the relationships between Hog1 and Tco2 or Ena1 in the cation-stress response of *T. asahii*. Hog1 in *C. neoformans* and SakA, a homolog of Hog1, in *A. fumigatus* show susceptibility to compounds that cause damage to the cell membrane^35,39,40^. SakA in *A. fumigatus* is required for the cell wall integrity signaling pathway and contributes to resistance against cell wall damage^39^. On the other hand, because the Hog1 signaling pathway and the cell wall integrity signaling pathway are not associated in *C. neoformans*, the *hog1* gene-deficient mutant does not show marked susceptibility to cell wall stress^38,40^. In *T. asahii*, Hog1 contributes to tolerance to cell membrane damage, but not to cell wall damage. Therefore, the function of Hog1 in *T. asahii* for the maintenance of cell membranes and cell walls is similar to that in *C. neoformans*. In *C. neoformans*, *C. albicans*, and *S. cerevisiae*, Hog1 plays crucial roles in the resistance to oxidative stress and ER stress^17,20,23,24,35,41–43^. The *hog1* gene-deficient *T. asahii* mutant showed sensitivity to oxidative stress, but not to ER stress. These observations suggest that Hog1 in *T. asahii* is an essential factor for oxidative stress resistance, and *T. asahii* possesses a mechanism involving Hog1-mediated oxidative stress resistance similar to other fungi such as *C. neoformans*. Hog1 in *C. neoformans* regulates the expression of oxidative stress-related factors such as catalase and mitochondrial manganese superoxide dismutase^54^. The increased sensitivity to H_2_O_2_ and menadione observed in the *hog1* gene-deficient *T. asahii* mutant may be due to a decrease in the activity of enzymes involved in the elimination of reactive oxygen species. Identification of the responsible enzymes regulated by Hog1 to eliminate reactive oxygen species in *T. asahii* will be an important future research aim. Hog1 in *C. neoformans* and *S. cerevisiae* contributes to resistance to DNA damage^17,35,44^. The sensitivity of the *hog1* gene-deficient *T. asahii* mutant against MMS and UV suggests that Hog1 is an essential factor for resistance to DNA mutations in *T. asahii*. We assumed that Hog1 in *T. asahii* is phosphorylated upon exposure to these stressors. The development of a specific antibody against *T. asahii* Hog1 is important to reveal the stress response mechanisms by phosphorylation.

*T. asahii* exhibits Hog1-mediated resistance to antifungal drugs. The *hog1* gene-deficient mutant in *C. albicans* shows sensitivities to AMPH-B and azole antifungal drugs^23^. In *C. neoformans*, the *hog1* gene-deficient mutant shows sensitivity to AMPH-B, but not to azole antifungal drugs^19,35,38^. Hog1 in *T. asahii* is involved in resistance against antifungal drugs. The combined use of Hog1 inhibitors and AMPH-B or azole antifungal agents might be effective in treating *T. asahii* infections.

*C. albicans* exhibits 2 forms of yeast and hyphae, and the hyphal elongation is negatively regulated by Hog1^20,21,23,25–28,48^. Because the *hog1* gene-deficient *T. asahii* mutant forms more hyphae than the parent strain, Hog1 might regulate hyphal formation in *T. asahii*. We hypothesized that Hog1 in *T. asahii* controls the expression of genes related to hyphal formation. Hyphal formation in *T. asahii* is induced by magnesium^55^. In *C. albicans*, magnesium deficiency activates Hog1^56^. Therefore, magnesium plays an important role in regulating Hog1 activation in fungi. In *T. asahii*, magnesium may suppress the Hog1 pathway and promote hyphal formation that is similar to the phenotype of the *hog1* gene-deficient mutant. Identifying the important genes involved in hyphal formation regulated by Hog1 is an important future challenge toward deepening our understanding of the morphological aspects of *T. asahii*.

The virulence of *T. asahii* against silkworms was reduced by *hog1* gene deficiency. Moreover, the number of viable cells in the *hog1* gene-deficient mutant decreased in the silkworm hemolymph. We speculate that *hog1* gene deficiency causes several stress sensitivities in host environments and results in a virulent phenotype in *T. asahii*. Therefore, the identification of host factors that induce Hog1-mediated stress responses is an important topic for future research.

Hog1 inhibitors may attenuate *T. asahii* virulence. However, these inhibitors may affect humans because Hog1 is highly conserved among eukaryotes. Therefore, the development of fungal Hog1-specific inhibitors is required. In calcineurin, which is highly conserved in eukaryotes like Hog1, a fungal-specific calcineurin inhibitor have been synthesized by focusing on the subtle structural differences between human calcineurin and fungal calcineurin^57^. We assumed that it might be possible to construct selective inhibitory compounds for fungal Hog1 based on structural differences, similar to the development of inhibitory compounds for calcineurin.

In conclusion, *T. asahii* controls stress response and virulence via Hog1. Hog1-mediated stress responses might contribute to antifungal drug resistance and virulence of *T. asahii* by facilitating its adaptation to the host environments. These findings suggest that Hog1 inhibitors, as anti-infectious agents in combination with antifungal drugs, may be useful in treating *T. asahii* infection.

## Methods

### Reagents

Nourseothricin was purchased from Jena Bioscience (Dortmund, Germany). Cefotaxime sodium, D-glucose, agar, NaCl, KCl, sorbitol, H_2_O_2_, DTT, MMS, and AMPH-B were purchased from Fujifilm Wako Pure Chemical Industries (Osaka, Japan). Hygromycin B, caffeine, FLCZ, VRCZ, and N-phenylthiourea were purchased from Tokyo Chemical Industry Co., Ltd. (Tokyo, Japan). Congo Red and menadione were purchased from Sigma-Aldrich (St. Louis, MO, USA). G418 was purchased from Enzo Life Science, Inc. (Farmingdale, NY, USA). Hipolypeptone was purchased from Nihon Pharmaceutical Co., Ltd. (Tokyo, Japan). Tunicamycin (TM) was purchased from Cayman Chemical Company (Ann Arbor, MI, USA). Sodium dodecyl sulfate (SDS) was purchased from Nippon Gene Co., Ltd. (Tokyo, Japan). Tween 80 was purchased from MP Biomedicals LLC (Santa Ana, MO, USA).

### Comparison of amino acid sequences of typical fungal Hog1 proteins

The amino acid sequences of Hog1 proteins in *T. asahii*, *C. neoformans*, *C. albicans*, and *S. cerevisiae*, and SakA protein in *A. fumigatus* were obtained from the Ensembl Fungi (http://fungi.ensembl.org/index.html). The Ensembl Fungi gene IDs of the proteins are shown in Supplementary Table 1. The functional domains of the proteins were estimated by the NCBI website (https://www.ncbi.nlm.nih.gov). The NCBI domain accession numbers of the proteins are shown in Supplementary Table 2. The phylogenic tree was constructed according to the maximum likelihood method using MEGA11 Software (https://www.megasoftware.net/). Amino acid sequence alignment was performed using MEGA11 Software.

### Culture of *T. asahii*

The *T. asahii* strain (MPU129 *ku70* gene-deficient mutant) used in this study was generated as previously reported^30^. The *T. asahii* MPU129 *ku70* gene-deficient mutant was grown on SDA (1% hipolypeptone, 4% dextrose, and 1.5% agar) containing G418 (100 μg/mL) and incubated at 27 °C for 2 days.

### Construction of the *hog1* gene-deficient *T. asahii* mutant and the revertant

The plasmid for gene-deficient *T. asahii* strains was constructed according to a previous report^34^. To generate the *hog1* gene-deficient strain, the 5′-UTR and 3′-UTR of the *hog1* gene were introduced into a pAg1-NAT1 vector^30^ using the infusion method (In-Fusion HD Cloning Kit, Takara, Shiga, Japan). To generate the revertant, the hygromycin phosphotransferase gene (*hph*) cassette and the *hog1* gene were introduced into pAg1-*hog1*(5′UTR)-*NAT1*-*hog1*(3′UTR). The primers used for PCR amplification of each DNA region are shown in Supplementary Table 3.

Gene transfer using electroporation was performed according to a previous report^33^. The 5′-UTR (*hog1*)-*NAT1*-3′-UTR (*hog1*) or 5′-UTR (*hog1*)-*hog1*-*hph*-3′-UTR (*hog1*) fragments were amplified by PCR with the primers shown in Supplementary Table 3. The *T. asahii* competent cells (40 µL) with the DNA fragments (90–180 ng) were added to a 0.2-cm gap cuvette (Bio-Rad Laboratories, Inc.) and electroporated (Time constant protocol: 1800 V, 5 ms) using a Gene Pulser Xcell (Bio-Rad Laboratories, Inc.). The cells were suspended by yeast peptone dextrose containing 0.6 M sorbitol and incubated at 27 °C for 3 h. After incubation, the cells were applied to SDA containing nourseothricin (300 µg/mL) or hygromycin B (300 µg/mL) and incubated at 27 °C for 3 days. The mutation into the genome of the candidates growing on the SDA containing drugs was confirmed by PCR with the primers shown in Supplementary Table 3. Information on the strains is provided in Table 3.

**Table 3:** *T. asahii* strains used in this study.

*T. asahii* strains	Relevant genotype	Background	References
MPU129 ∆*ku70* (Parent strain)	*ku70*::*nptII*	MPU129	Matsumoto et al.^30^
∆*hog1*	*ku70*::*nptII*, *hog1*::*NAT1*	MPU129 ∆*ku70*	This study
Hog1 (revertant)	*ku70*::*nptII*, *NAT1*::*hog1*, *hph*	MPU129 ∆*ku70* ∆*hog1*	This study

### Temperature sensitivity test

The temperature sensitivity test was performed according to a previous report with slight modifications^34^. The *T. asahii* cells grown on SDA containing cefotaxime (100 μg/mL) at 27 °C for 1 day were suspended in physiologic saline solution with 0.01% Tween 80 and filtered through a 40-μm cell strainer (Corning Inc., Corning, NY, USA). The *T. asahii* cell suspension was adjusted to 1–1.2 on absorbance at 630 nm. Series of a tenfold dilution of the fungal suspension were prepared using saline. Five microliters of each cell suspension were spotted on the SDA. The agar plates were incubated at 27, 37, or 40 °C for 48 h, and photographs were obtained.

For growth on liquid medium, Sabouraud liquid medium (1% hipolypeptone, 4% dextrose) was used in this study. Cell suspensions of the *T. asahii* strains were prepared with Sabouraud liquid medium. The *T. asahii* suspensions (A_630_ = 0.005) were incubated at 27 °C, 37 °C, or 40 °C for 4 days and absorbance at 630 nm was measured using a microplate reader (iMark microplate reader; Bio-Rad Laboratories Inc., Hercules, CA, USA).

### Drug sensitivity test using agar plate

The drug sensitivity test was performed according to a previous report with slight modifications^34^. Series of a tenfold dilution of the fungal suspension were prepared in the same manner as for the temperature sensitivity test described above. Five microliters of each cell suspension were spotted on the SDA containing NaCl (1.2 M), KCl (1.5 M), caffeine (0.65 mg/mL), Congo red (400 µg/mL), SDS (0.008%), H_2_O_2_ (3 mM), menadione (60 µM), TM (1 µg/mL), DTT (12 mM), MMS (0.05%), AMPH-B (0.4 µg/mL), FLCZ (18 µg/mL), or VRCZ (0.2 µg/mL). Each agar plate was incubated at 27 °C for 24–48 h, and photographs were obtained.

### UV sensitivity test

Series of a tenfold dilution of the fungal suspension were prepared in the same manner as for the temperature sensitivity test described above. Five microliters of each cell suspension was spotted on the SDA. The SDA plate was exposed to UV light using a UV crosslinker (CX-2000; UVP LLC, Upland, USA). Each agar plate was incubated at 27 °C for 24 h, and photographs were obtained.

### MIC determination

The MIC values were determined by a micro-liquid dilution method (CLSI, M27-A3) using antifungal susceptibility test plates (Yeast-like fungus DP ‘Eiken’; Eiken Chemical Co., Ltd., Tokyo, Japan)^58^. The *T. asahii* cells on SDA containing cefotaxime (100 μg/mL) was suspended in physiologic saline solution with 0.01% Tween 80. The *T. asahii* cell suspension was adjusted to McFarland 1. Each fungal suspension was diluted 20-fold in saline, and then diluted 100-fold in RPMI 1640 medium (Thermo Fisher Scientific Inc., MA, USA) containing 0.165 M MOPS (FUJIFILM Wako Pure Chemical Corporation, Osaka, Japan) (RPMI MOPS) (pH 7.0). One hundred microliters of each cell suspension was applied to wells of the antifungal susceptibility test plates. The test plates were incubated at 37 °C for 48 h. The MIC values were determined from the results of two independent experiments.

### Observation of *T. asahii* morphology

The morphology observation was performed according to a previous report^34^. The *T. asahii* cells were suspended in physiologic saline solution with 0.01% Tween 80 and filtered through a 40-μm cell strainer (Corning Inc.). The cell suspension (10 µL) adjusted to 1 on absorbance at 630 nm was added to Sabouraud dextrose liquid medium (90 µL) or harvested silkworm hemolymph (90 µL). The incubated solution at 27 °C for 48 h was placed on glass slides and covered by glass coverslips. Samples were examined with bright light under a microscope (CH-30; Olympus, Tokyo, Japan). The pictures were randomly obtained. The morphology of *T. asahii* was determined according to a previous report^59^. The numbers of the following 3 types of cells were counted: yeast (spherical cells including budding yeasts and may include blastoconidia); arthroconidia (≥ 3 round or rectangular cells connected with their interiors separated by septa); and hyphae (cells elongated to a length of at least 2 yeast cells possessing no septa).

### Silkworm infection experiments

Silkworm infection experiments were performed according to a previous report with slight modifications^30^. Eggs of silkworms (Hu·Yo × Tukuba·Ne) were purchased from Ehime-Sanshu Co, Ltd. (Ehime, Japan). Silkworm fifth instar larvae were fed the artificial diet (Silkmate 2S; Ehime-Sanshu Co., Ltd.) overnight. The *T. asahii* grown on SDA plates incubated for 1 day at 27 °C was suspended in physiologic saline solution with 0.01% Tween 80 and filtered through a 40-μm cell strainer (Corning Inc.). A 50-µL suspension of *T. asahii* cells was administered to the silkworms and the silkworms injected with *T. asahii* cells were incubated at 37 °C.

### LD_50_ measurement

The dose of *T. asahii* required to kill half of the silkworms (LD_50_) was determined according to a previous report with slight modifications^30^ *T. asahii* strains (1 × 10^2^ to 5.5 × 10^4^ cells/50 µL) were injected into the silkworm hemolymph and the silkworms were incubated at 37 °C. Survival of the silkworms (n = 4/group) at 72 h was recorded. The LD_50_ was determined from the combined data of 2 independent experiments by a simple logistic regression model using Prism 9.1.2 (GraphPad Software, LLC, San Diego, CA, USA, https://www.graphpad.com/scientific-software/prism/).

### Cell viability of *T. asahii* in silkworm hemolymph

The *T. asahii* cells grown on SDA containing cefotaxime (100 μg/mL) were suspended in physiologic saline solution with 0.01% Tween 80 and filtered through a 40-μm cell strainer (Corning Inc.). Ten microliters of each cell suspension (A_630_ = 1–1.5) was added to 90 µL of filter-sterilized silkworm hemolymph. The cell suspensions were cultured at 37 °C for 4 days and collected every 24 h. The collected cell suspensions were diluted 2000-fold with physiologic saline and 100 μL was spread on SDA. The number of colonies on the SDA after 1 day incubation were counted.

### Statistical analysis

All experiments were performed at least twice and representative results are shown. The significance of differences between groups in the silkworm infection experiments in Fig. 7a was calculated by the log-rank test based on curves determined using the Kaplan–Meier method with Prism 9.1.2. *P* < 0.05 was considered significant. Statistically significant differences between 0 and 4 days in Fig. 8a were evaluated using Student’s *t*-test. Statistically significant differences between groups in Fig. 8b were evaluated using Tukey’s test.

### Supplementary Information

Supplementary Information.

## Data Availability

The datasets generated in the present study are available from the corresponding author upon reasonable request.
