# Supplementary material for: Hog1-mediated stress tolerance in the pathogenic fungus Trichosporon asahii
Source: Sci Rep. 2023 Aug 19;13:13539. doi: 10.1038/s41598-023-40825-y (PMC10439922; doi:10.1038/s41598-023-40825-y)
Supplement: Supplementary file 1 — Supplementary Information. [file 41598_2023_40825_MOESM1_ESM.pdf]

Supplementary information

Hog1-mediated stress tolerance in the pathogenic fungus *Trichosporon asahii*

Yasuhiko Matsumoto[1, 2*], Yu Sugiyama[1, 2*], Tae Nagamachi[1], Asami Yoshikawa[1], and Takashi Sugita[1]

[1]Department of Microbiology, Meiji Pharmaceutical University, 2-522-1, Noshio, Kiyose, Tokyo 204-8588, Japan.

[2]These authors contributed equally: Yasuhiko Matsumoto and Yu Sugiyama.

*Address correspondence to: Dr. Yasuhiko Matsumoto, Department of Microbiology, Meiji Pharmaceutical University, 2-522-1, Noshio, Kiyose, Tokyo 204-8588, Japan, Tel: +81-42-495-8745, e-mail: ymatsumoto@my-pharm.ac.jp.

Supplementary table 1 Ensembl Fungi gene IDs for Hog1 proteins in the phylogenetic tree

Species	Definition	Gene ID	Size (aa)	Query Cover (%)	Percent Identical (%)
Trichosporon asahii	mitogen-activated protein kinase [*Trichosporon asahii* var. *asahii* CBS 8904]	A1Q2_01686	368	100	100
Cryptococcus neoformans	CMGC/MAPK/P38 protein kinase [*Cryptococcus neoformans* var. *grubii* H99]	CNAG_01523	365	99	96.71
Aspergillus fumigatus	MAP kinase SakA [*Aspergillus fumigatus* Af293]	AFUA_1G12940	366	98	85.16
Saccharomyces cerevisiae	mitogen-activated protein kinase HOG1 [*Saccharomyces cerevisiae* S288C]	GI526_G0003907	435	92	80.12
Candida albicans	mitogen-activated protein kinase HOG1 [*Candida albicans* SC5314]	I503_01784	377	95	76.34

Supplementary table 2 NCBI domain accession number for Hog1 proteins

Domain name	NCBI accession	Description	Species	Interval (aa)
STKc_Sty1_Hog1	cd07856	Catalytic domain of the Serine/Threonine Kinases, Fungal Mitogen-Activated Protein Kinases Sty1 and Hog1	*Trichosporon asahii*<br>*Cryptococcus neoformans*<br>*Aspergillus fumigatus*<br>*Saccharomyces cerevisiae*<br>*Candida albicans*	9-336<br>9-336<br>9-336<br>12-339<br>(-)
PKc-like super family	cl21453	Protein Kinases, catalytic domain	*Trichosporon asahii*<br>*Cryptococcus neoformans*<br>*Aspergillus fumigatus*<br>*Saccharomyces cerevisiae*<br>*Candida albicans*	9-336<br>9-336<br>9-336<br>12-339<br>12-342

Supplementary table 3 Primers used in this study

Primers	Nucleic acid sequence
<u>pAg1-*hog1*(5'UTR)-*NAT1*-*hog1*(3'UTR) for cloning</u>	
**pAg1-*hog1*(5'UTR)-*NAT1* (1st cloning)**	
F *NAT1* for *hog1*(5'UTR)	GCTGCGAGGATGTGAGCTGGAGAGC
R pAg1 for *hog1*(5'UTR)	CTCCGCTCATGATCAGATTGTCGTTTCCCG
F *hog1*(5'UTR)	ATCTGATCATGAGCGGAGCGCAGGTGGTGACGACTGGACGGATCTCG
R *hog1*(5'UTR)	AGCTCACATCCTCGCAGCGGGATGGAGGCAGGAGGCTTGGGAAAGATGC
**pAg1-*hog1*(5'UTR)-*NAT1*-*hog1*(3'UTR) (2nd cloning)**	
F pAg1 for *hog1*(3'UTR)	GAAAACCCTGGCGTTACCCAACTTAATCG
R *NAT1* for *hog1*(3'UTR)	GAAGAGATGTAGAAACTAGCTTCCTGGTTTCAGAG
F *hog1*(3'UTR)	TAGTTTCTACATCTCTTCGTGTTGACTTGAGTGTGCACGGTGCTCGG
R *hog1*(3'UTR)	GGTAACGCCAGGGTTTTCGCTCGAGCAGAAGGTTCTGCAGCTCGGTG
**Amplification of *hog1* cassette for electroporation**	
F *hog1*-cassette	ATCTGATCATGAGCGGAGCGCAGGTGGTGACGACTGGACGGATCTCG
R *hog1*-cassette	GGTAACGCCAGGGTTTTCGCTCGAGCAGAAGGTTCTGCAGCTCGGTG
<u>pAg1-*hog1*(5'UTR)-*hog1*-*hph*-*hog1*(3'UTR) for cloning</u>	
**pAg1-*hog1*(5'UTR)-*hph*-*hog1*(3'UTR) (1st cloning)**	
F 3'UTR-pAg1-5'UTR for *hph*	GTGTTGACTTGAGTGTGCACGGTGCTCGG
R 3'UTR-pAg1-5'UTR for *hph*	GGGATGGAGGCAGGAGGCTTGGGAAAGATGC
F *hph*	GCCTCCTGCCTCCATCCCGGGCCCCCTGCGAGGATG
R *hph*	GCACACTCAAGTCAACACGGATCCGAAGAGATGTAGAAAC
**pAg1-*hog1*(5'UTR)-*hog1*-*hph*-*hog1*(3'UTR) (2nd cloning)**	
F *hph*-3'UTR-pAg1-5'UTR for *hog1*	GGGCCCCCTGCGAGGATG
R *hph*-3'UTR-pAg1-5'UTR for *hog1*	GGGATGGAGGCAGGAGGCTTGGGAAAGATGC
F *hog1*	GCCTCCTGCCTCCATCCCACTCTCCCCTTGTCCAGCAATCCAACGCTTGC
R *hog1*	CATCCTCGCAGGGGGCCCCTAAGCGGCCGGGGCAGCGGC
<u>pUC19-*hog1*(5'UTR)-*hog1*-*hph*-*hog1*(3'UTR) for cloning</u>	
**pUC19-*hog1*(5'UTR)-*hog1*-*hph*-*hog1*(3'UTR) (1st cloning)**	
F 5'UTR-*hog1*-*hph*-3'UTR for pUC19	ATCTGATCATGAGCGGAGCGCAGGTGGTGACGACTGGACGGATCTCG
R 5'UTR-*hog1*-*hph*-3'UTR for pUC19	GGTAACGCCAGGGTTTTCGCTCGAGCAGAAGGTTCTGCAGCTCGGTG
F pUC19	AGAACCTTCTGCTCGAGCCAGAGGTTTTCACCGTCATCACCGAAACGC
R pUC19	CAGTCGTCACCACCTGCGGGCGAGCGGTATCAGCTCACTCAAAGGC
**pUC19-*hog1*(5'UTR)-*hog1*-*hph*-*hog1*(3'UTR) (2nd cloning)**	
F *hph*-3'UTR-pUC19-5'UTR-*hog1* for *hog1* (terminator)	GGGCCCCCTGCGAGGATG
R *hph*-3'UTR-pUC19-5'UTR-*hog1* for *hog1* (terminator)	CTAAGCGGCCGGGGCAGCG
F *hog1* (terminator)	GCTGCCCCGGCCGCTTAGAGGATTTGAGTCGAGGTTCTGATCACTGTC
R *hog1* (terminator)	CATCCTCGCAGGGGGCCCATCCTCTACCTGTATCCCGGGTCCC
**Amplification of *hog1* revertant cassette for electroporation**	
F *hog1* revertant-cassette	GTCCTGTCGGGTTTCGCCACCTCTGAC

R *hog1* revertant-cassette	GGGTTCCGCGCACATTTCCCCGAAAAG
**Primers-1 for *hog1* genotyping**	
F *hog1* gene ORF	CGGTTCTGAGCAAGAGGACATACCGTGAGCTC
R *hog1* gene ORF	GCGAAGATACATCCAGTGCTCCAGATGTCGACG
**Primers-2 for *hog1* genotyping**	
F *hog1* gene locus	ATCTGATCATGAGCGGAGCGCAGGTGGTGACGACTGGACGGATCTCG
R *hog1* gene locus	GGTAACGCCAGGGTTTTCGCTCGAGCAGAAGGTTCTGCAGCTCGGTG
**Primers-3 for *hog1* genotyping**	
F *hog1* gene outside 1	GGACGGCGAGCAGGCGCTCTACATGAGC
R *hog1* gene outside 1	CAGCACGGCTCTTGTCGCCTTCAGAGCC
**Primers-4 for *hog1* genotyping**	
F *hog1* gene outside 2	CAGGGTCGATGCGACGCAATCGTCCGATCC
R *hog1* gene outside 2	CAGCACGGCTCTTGTCGCCTTCAGAGCC

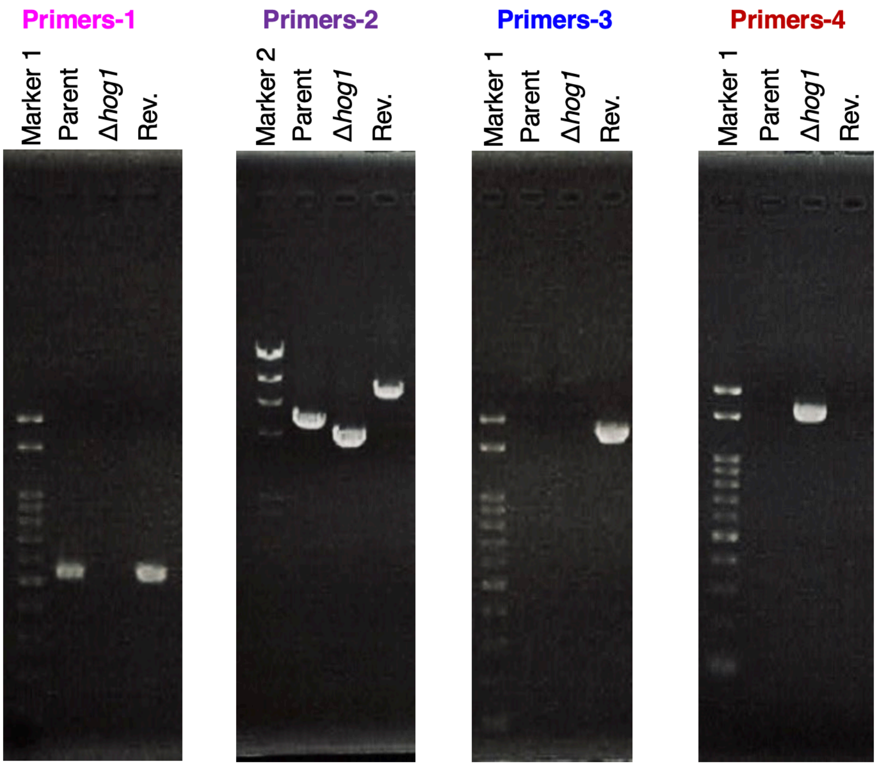

Supplementary Fig. S1 Full-length blots of Figure 1d. Marker 1: Gene Ladder 100 (Nippon Gene Co., Ltd., Tokyo, Japan), Maker 2: OneSTEP Marker 1(λ /HindIII digest) (Nippon Gene Co., Ltd., Tokyo, Japan).

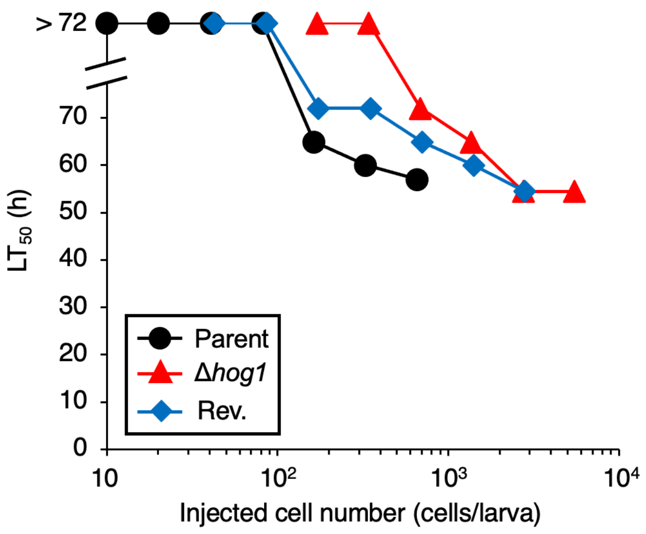

Supplementary Fig. S2 LT_{50} values of the parent, *hog1* gene-deficient, and revertant *T. asahii*.

Number of surviving silkworms at 37˚C was determined for 72 h after administration of the fungal cells (1×10^2 to 5.5×10^4 cells/larva) into the silkworm hemolymph. n = 4/group. The half-maximal lethal time (LT_{50}) is the time required to kill half the animals in a group.
